# Are relative educational inequalities in multiple health behaviors widening? A longitudinal study of middle-aged adults in Northern Norway

**DOI:** 10.3389/fpubh.2023.1190087

**Published:** 2023-08-22

**Authors:** Ana Silvia Ibarra-Sanchez, Gang Chen, Torbjørn Wisløff

**Affiliations:** ^1^Department of Community Medicine, The Arctic University of Norway, Tromsø, Norway; ^2^Centre for Health Economics, Monash University, Melbourne, VIC, Australia; ^3^Health Services Research Unit, Akershus University Hospital, Lørenskog, Norway

**Keywords:** educational inequality, health behavior, socioeconomic disparities, health guidelines, the Tromsø Study

## Abstract

**Introduction:**

Educational inequality in multiple health behaviors is rarely monitored using data from the same individuals as they age. The aim of this study is to research changes in relative educational inequality in multiple variables related to health behavior (smoking, physical activity, alcohol intake, and body mass index), separately and collectively (healthy lifestyle), among middle-aged adults living in Northern Norway.

**Methods:**

Data from adult respondents aged 32–87 in 2008 with repeated measurements in 2016 (*N* = 8,906) were drawn from the sixth and seventh waves of the Tromsø Study. Logistic regression was used to assess the relative educational inequality in the variables related to health behavior. The analyses were performed for the total sample and separately for women and men at both baseline and follow-up.

**Results:**

Educational inequality was observed in all the variables related to health behavior at baseline and follow-up, in both men and women. Higher levels of educational attainment were associated with healthier categories (non-daily smoking, physical activity, normal body mass index, and a healthy lifestyle), but also with high alcohol intake. The prevalence of daily smoking and physical inactivity decreased during the surveyed period, while high alcohol intake, having a body mass index outside of the normal range and adhering to multiple health recommendations simultaneously increased. The magnitude of relative educational inequality measured at baseline increased at the follow-up in all the variables related to health behavior. Differences were larger among women when compared to men, except in physical inactivity.

**Conclusion:**

Persistent and increasing relative disparities in health behavior between the highest education level and lower education levels are found in countries with well-established and comprehensive welfare systems like Norway. Addressing these inequalities is essential for reducing both the chronic disease burden and educational disparities in health.

## 1. Introduction

Health inequalities are often observed along a social gradient, where a stepwise or linear decrease in health comes with decreasing socioeconomic position (1).

In Norway, not only are there large socioeconomic inequalities in both mortality and self-reported health (2–4), but these differences have also been widening (3). The disparities are substantial, especially between educational groups (5, 6). This observation is of considerable interest in the Nordic countries, due to progressive tax regimes that redistribute income and comprehensive welfare systems that subsidize healthcare and education across all levels, from primary to tertiary education (7, 8). Thus, large and persistent health disparities pose a challenge to welfare states, suggesting the need for understanding the determinants underlying the social gradient in health.

Health behaviors, such as smoking, alcohol intake, physical activity, and diet are also unevenly distributed across socioeconomic groups, and they contribute to the social gradient in cardiometabolic disease and mortality (9). Moreover, cross-sectional studies have reported variation in health behavior factors based on diverse indicators of socioeconomic position (6, 10, 11). The dynamics of socioeconomic inequalities in health outcomes are well-documented. However, there is less knowledge about the dynamics of social inequalities in multiple factors of health behavior, particularly from research contributions following the same study population over time. Follow-up studies are advantageous regarding the establishment of the temporal occurrence and directionality of both the exposure and the effects of this exposure (12, 13).

Educational inequalities in various health behavior factors have been observed in different longitudinal studies among Norwegian populations (14, 15). Contrasting trends have been reported among the studies focusing on socioeconomic inequality dynamics in chronic disease mediators that include behavioral factors. For instance, a 15 years follow-up study that followed the respondents from childhood to adulthood found persisting socioeconomic inequalities in the consumption of sweetened beverages (16). The participants with higher educational levels in adulthood and higher educational intentions during adolescence had a significantly lower frequency of consumption of sweetened beverages, and these inequalities neither widened nor narrowed over time. In the late 1970 and 1980's, a follow-up study of adults aged 35–49 years old found higher education to be associated with lower body mass index (BMI) and less prevalence of smoking (17). This educational inequality in smoking decreased among men and women, whereas in terms of BMI, it persisted among women and decreased for men. More recently, a longitudinal study with repeated cross-sections of adults aged 40–59 years old found that inequality in smoking by education level has been widening (18) with higher prevalence among those with primary and lower secondary school. Regarding BMI, a study of adults aged 30–69 years reported a higher prevalence of obesity in groups with lower education (19). This study revealed that the relative inequality remained unchanged, whereas the absolute inequality increased.

The existing research contributions provide important insight into trends of social inequalities in single health behavior factors as well as in pairs of these factors. Nevertheless, the dynamics of the socioeconomic gradient in health behavior assessed by multiple health behavior factors within the same study population are not well-understood. Addressing multiple health behavior factors is important due to the higher risk for chronic diseases and all-cause mortality associated with a higher number of unhealthy behavior factors (20–23). Furthermore, following the same individuals over time allows for studying exposure effect patterning (12). The pattern of change is key due to the variable nature of an individual's health behavior exposure, given that changes in their behavior factors can arise as they transition through middle-age. Therefore, this article aims to research changes in relative educational inequality in four measures related to health behavior (daily smoking, physical inactivity, high alcohol intake, and BMI) using data from respondents with repeated measurements in 2008 and 2016, from a population sample of adult women and men living in Northern Norway.

## 2. Methods

### 2.1. Study population and sample

Data were drawn from the sixth and seventh waves of the Tromsø Study, an epidemiological, prospective study of health conditions among adults residing in the municipality of Tromsø in Northern Norway. It consists of seven waves (labeled Tromsø 1–7) that were conducted from 1974 to 2016. The participants' information was obtained through questionnaires, screening visits, and several follow-up studies (24, 25).

Respondents' data on three health behavior factors (smoking, alcohol intake, and physical activity) and education level were retrieved from the questionnaires, while BMI was calculated from the participants' height and weight which were measured objectively at the time of each survey. Health behavior data were standardized by the sixth wave of the Tromsø Study (Tromsø 6), for subsequent waves to gather information on health behavior in the same manner. Thus, to monitor educational inequality in the prevalence of health behavior factors between 2008 and 2016, the study sample was comprised of adult respondents aged 32–87 in 2008 who participated in both the sixth and seventh waves of the prospective study (*N* = 8,906). The characteristics of the study sample's participants are presented in Supplementary Table 1.

This study was approved by the Regional Committees for Medical and Health Research Ethics (REK; ID: REK 2019/607). Informed consent for present and future data usage for research purposes was obtained from all study participants.

### 2.2. Measures

This study focuses on four factors related to health behavior (smoking, alcohol intake, physical activity, and BMI). A dichotomized variable was created for each behavior factor to research the adherence to current health recommendations, separately for each factor as well as collectively; that is, avoiding all forms of tobacco and high alcohol intake (more than 14 units per week for men and seven units per week for women) and maintaining a BMI within the recommended limits (18.5–24.9 kg/m^2^) by engaging in physical activity for at least 150 min per week (26–30).

#### 2.2.1. Daily smoking

The coding of the smoking variable was based on the question: “Do/did you smoke daily? (a) Yes, now (b) Yes, previously (c) Never.” Responses marked as “Yes, previously” or “Never” were coded as non-daily smokers, and “Yes, now” as daily smokers.

#### 2.2.2. Alcohol intake

Units per week of alcohol intake were calculated based on two questions regarding units and frequency of consumption. The responses to these questions were reconciled by the survey as follows: (1) “How many units of alcohol (one beer, glass of wine, or another beverage) do you usually drink when you consume alcohol?” (a) One to two = 1.5, (b) Three to four = 3.5, (c) Five to six = 5.5, (d) Seven to nine = 8, and (e) 10 or more = 12. (2) “How often do you usually drink alcohol?” (a) Never = 0, (b) Monthly or less frequently = 0.25, (c) Two to four times a month = 0.75, (d) Two to three times a week = 2.5, and (e) Four or more times a week = 5.5. The variable of units per week was created by multiplying the units times the frequency of intake (units per week = units × frequency). According to health recommendations, high alcohol intake is set at over 14 units per week for men and more than seven units per week for women (28, 29).

#### 2.2.3. Physical activity

The variable of physical activity was coded based on two questions regarding the duration and frequency of physical activity. The responses to these questions were reconciled by the survey as follows: (1) “On average, how long do you exercise for?” (a) <15 min = 10, (b) 15–29 min = 22, (c) 30–60 min = 45, (d) More than 1 h = 90. (2) “How often do you exercise (i.e., walking, skiing, swimming, or training any sports)?” (a) Never = 0, (b) Less than once a week = 0.5, (c) Once a week = 1, (d) Two to three times per week = 2.5, and (e) Approximately every day = 5. The variable of minutes per week of physical activity was created by multiplying the duration times the frequency (minutes per week = duration × frequency). Respondents were classified as having either <150 or 150 min or more of physical activity per week, according to current health recommendations (28, 30, 31).

#### 2.2.4. BMI

BMI was calculated using the objective measurement of the participant's height and weight, BMI = weight [kg] ÷ [height^2^] [m^2^]. A normal BMI is within the range of 18.5–24.9 kg/m^2^ (27). Respondents whose BMI was within this range were coded as having normal BMI, and those with a BMI outside of the normal range were coded as having abnormal BMI.

#### 2.2.5. Healthy lifestyle

A variable was created to represent the share of respondents that adhered to all the guidelines regarding the four health behavior factors; that is, participants who simultaneously do not smoke, have a low alcohol intake, exercise 150 min or more every week, and have a normal BMI.

#### 2.2.6. Education

Education level was obtained from the question: “What is the highest education level you have completed? (a) Primary/partly secondary education (up to 10 years of schooling), (b) Upper secondary education (minimum of 3 years), c) Tertiary education, short: university level, <4 years, (d) Tertiary education, long: university level, 4 years or more.” For simplicity, education levels are referred to as education levels 1–4 in the remaining text, where education level 1 indicates the lower educational level and level 4 represents the highest educational level.

### 2.3. Statistical analysis

Prevalence rates and odds ratios (ORs) of educational differences were calculated for daily smoking, high alcohol intake, physical inactivity, abnormal BMI, and the healthy lifestyle variable. Odds ratios were estimated by logistic regression at the baseline (model 1) and at the follow-up (model 2). To examine the variation in the educational differences between the two-time points, the third regression model (model 3) estimated the educational differences ORs at the follow-up controlling for each health behavior variable at baseline. Complete case analyses were performed, and potential confounding was accounted for by controlling for sex and age. All analyses were performed for the total sample and separately for women and men, due to previous research suggesting sex heterogeneity in both health behavior prevalence (32, 33) and health behavior inequality measures (33, 34). The analyses were computed using RStudio version 2022.07.1.

## 3. Results

### 3.1. Total cohort sample

#### 3.1.1. Daily smoking

**Figure 1:**
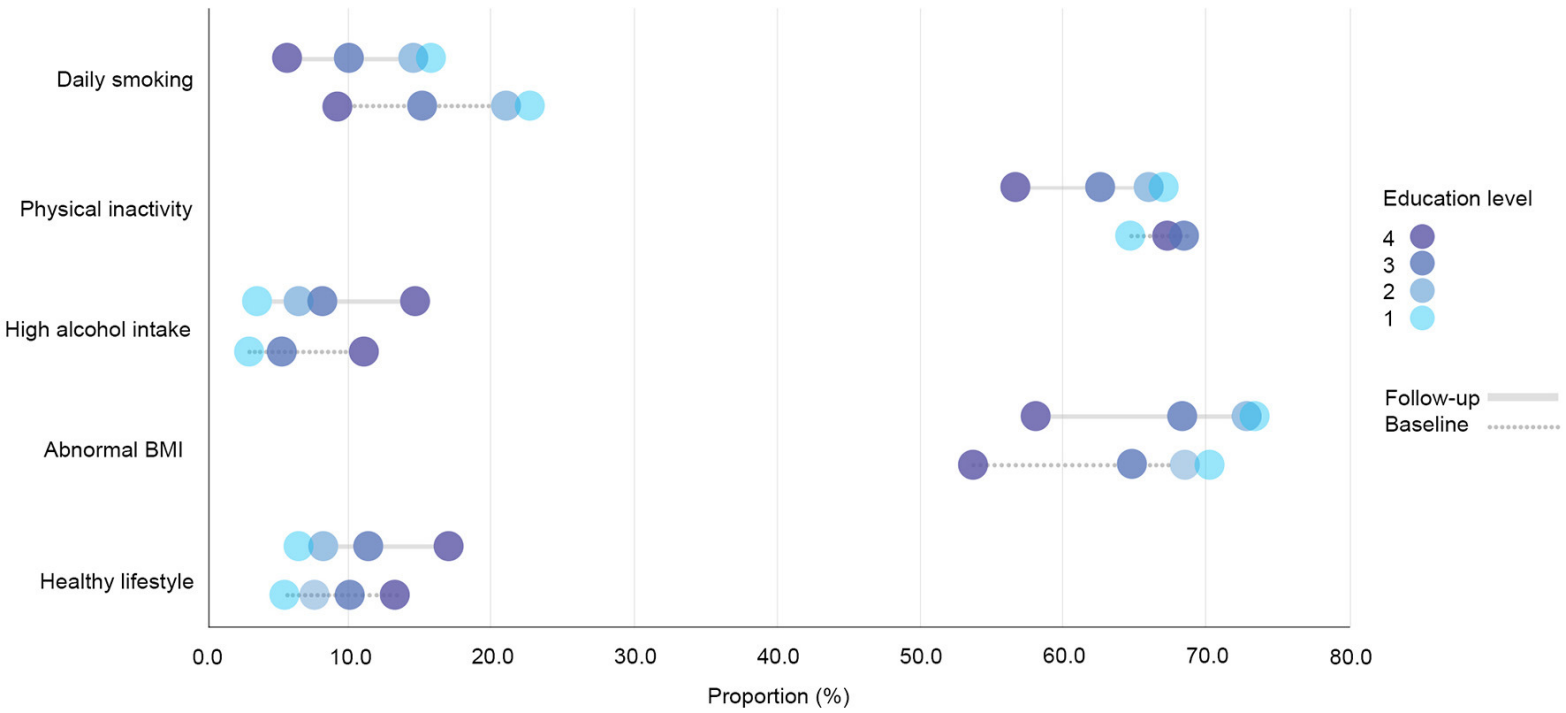
Prevalence rates of health behavior variables in the total cohort sample (*N* = 8,906) by education level at the baseline and the follow-up. Education level 1: Primary/partly secondary education; Education level 2: Upper secondary education; Education level 3: University (<4 years); Education level 4: University (more than 4 years).

**Figure 2:**
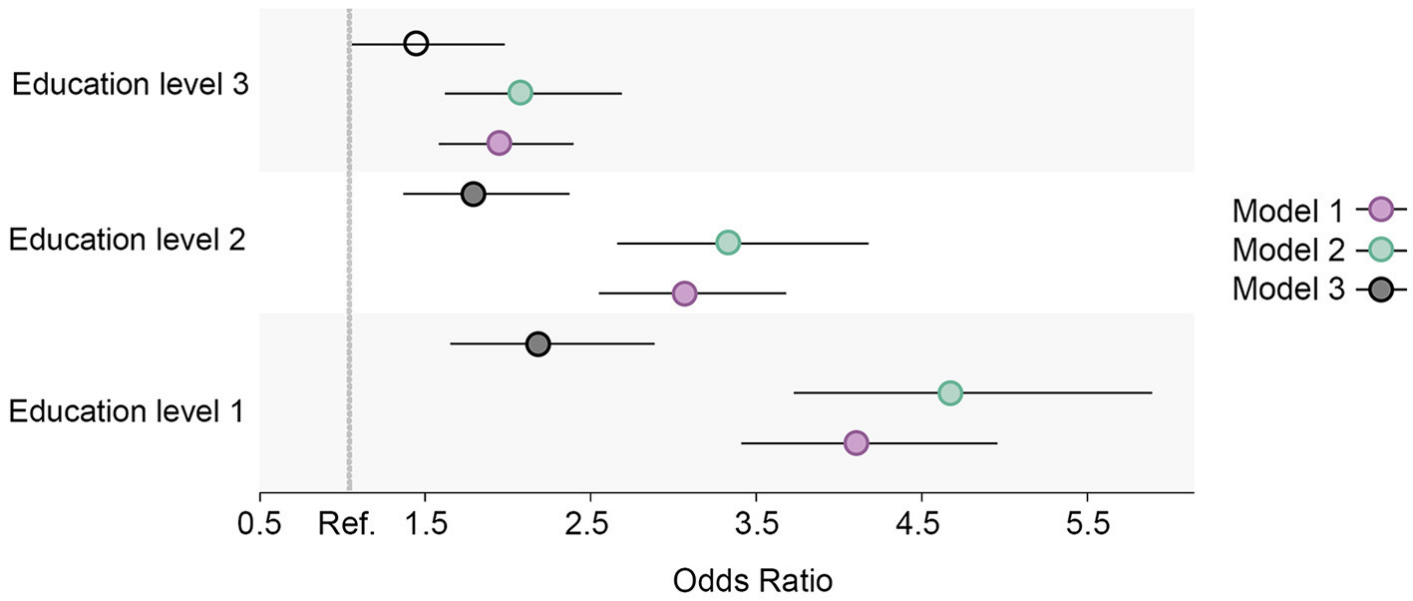
Educational differences in daily smoking in the total cohort sample, adjusted for age and sex. Model 1: Effect of education on daily smoking at the baseline; Model 2: Effect of education on daily smoking at the follow-up; Model 3: Effect of education on daily smoking at the follow-up controlled for daily smoking at baseline. Education level 1: Primary/partly secondary education; Education level 2: Upper secondary education; Education level 3: University (<4 years); Education level 4: University (more than 4 years). Reference category: Education level 4 [university (4 years or more)].

The overall prevalence of daily smoking in the study sample decreased throughout the surveyed period and was highest among respondents with the two lowest education levels at both time points (Figure 1). After adjusting for age and sex in the logistic regression model (Figure 2), clear educational differences were observed at both time points (Models 1 and 2). The regression estimates followed a gradient pattern, where the OR gradually increased with lower education levels. The gradient pattern was also observed at the follow-up, and the regression estimates were more pronounced, indicating that differences widened from baseline to the follow-up. The latter was confirmed with the clear gradient shown in the analysis that adjusted for baseline daily smoking (Model 3).

#### 3.1.2. Physical inactivity

**Figure 3:**
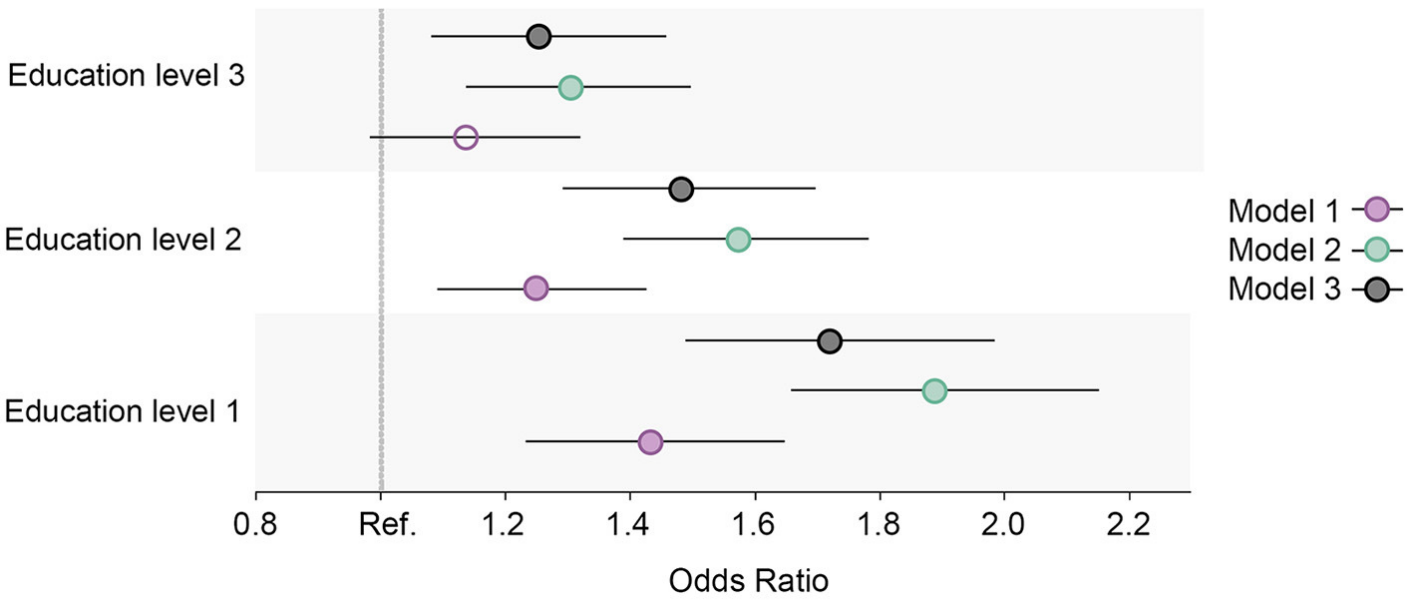
Educational differences in physical inactivity in the total cohort sample, adjusted for age and sex. Model 1: Effect of education on physical inactivity at the baseline; Model 2: Effect of education on physical inactivity at the follow-up; Model 3: Effect of education on physical inactivity at the follow-up controlled for physical inactivity at baseline. Education level 1: Primary/partly secondary education; Education level 2: Upper secondary education; Education level 3: University (<4 years); Education level 4: University (more than 4 years). Reference category: Education level 4 [university (4 years or more)].

The prevalence of physical inactivity in the study sample decreased during the surveyed period yet remained high (>60%; Figure 1). However, the group with the lowest education level was the only education group that showed an increase in the share of respondents with physical inactivity at the follow-up. The baseline regression model showed no distinct differences in the proportion of physical inactivity between the two highest education levels, yet there was a difference between the highest level and the two lowest education levels (Figure 3). A more pronounced gradient pattern was observed at the follow-up, with the size of the OR having grown and increased gradually in agreement with lowering education levels. When controlling for physical inactivity at the baseline (Model 3), a clear gradient was also observed, thereby confirming the increase in educational inequality between the highest education level and the other three levels.

#### 3.1.3. High alcohol intake

**Figure 4:**
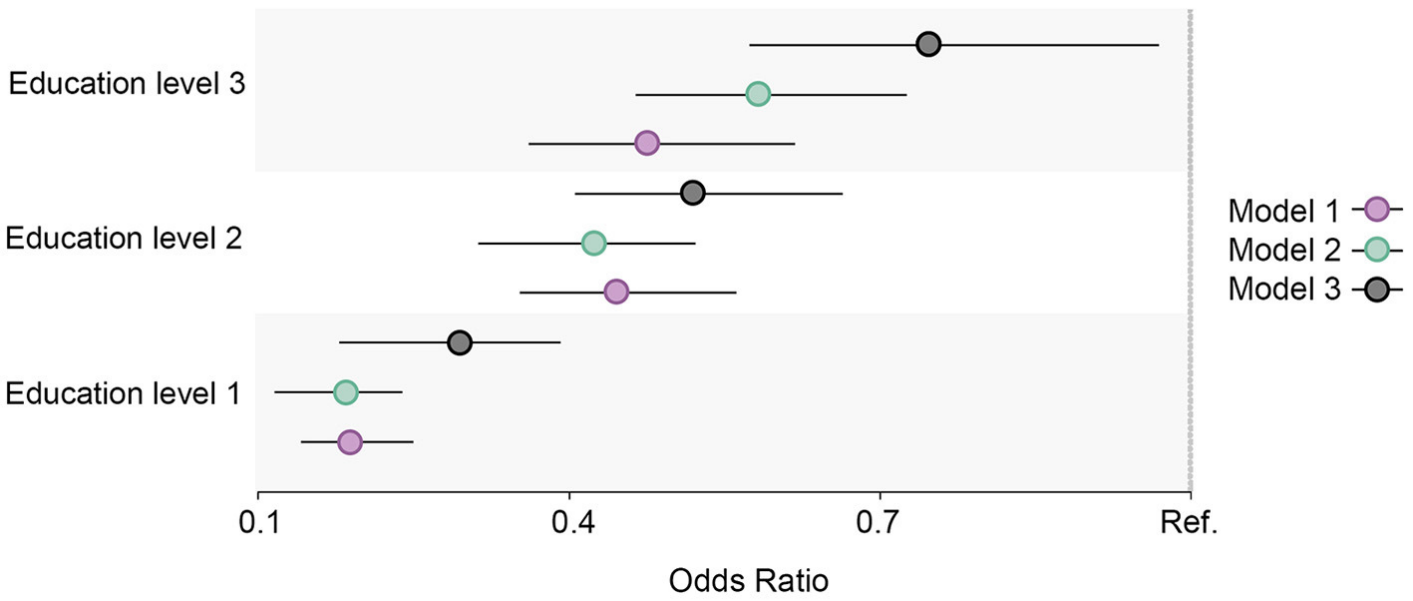
Educational differences in high alcohol intake in the total cohort sample, adjusted for age and sex. Model 1: Effect of education on high alcohol intake at the baseline; Model 2: Effect of education on high alcohol intake at the follow-up; Model 3: Effect of education on high alcohol intake at the follow-up controlled for high alcohol intake at baseline. Education level 1: Primary/partly secondary education; Education level 2: Upper secondary education; Education level 3: University (<4 years); Education level 4: University (more than 4 years). Reference category: Education level 4 [university (4 years or more)].

High alcohol intake increased and was highest among those with the highest education level at both measurement points (Figure 1). The regression estimates indicated that a high alcohol intake followed a reverse gradient pattern, where the OR decreased with a lower level of education (Figure 4). The clear gradient shown in the second and third regression models indicated that educational inequality increased substantially between the two-time points.

#### 3.1.4. Abnormal BMI

**Figure 5:**
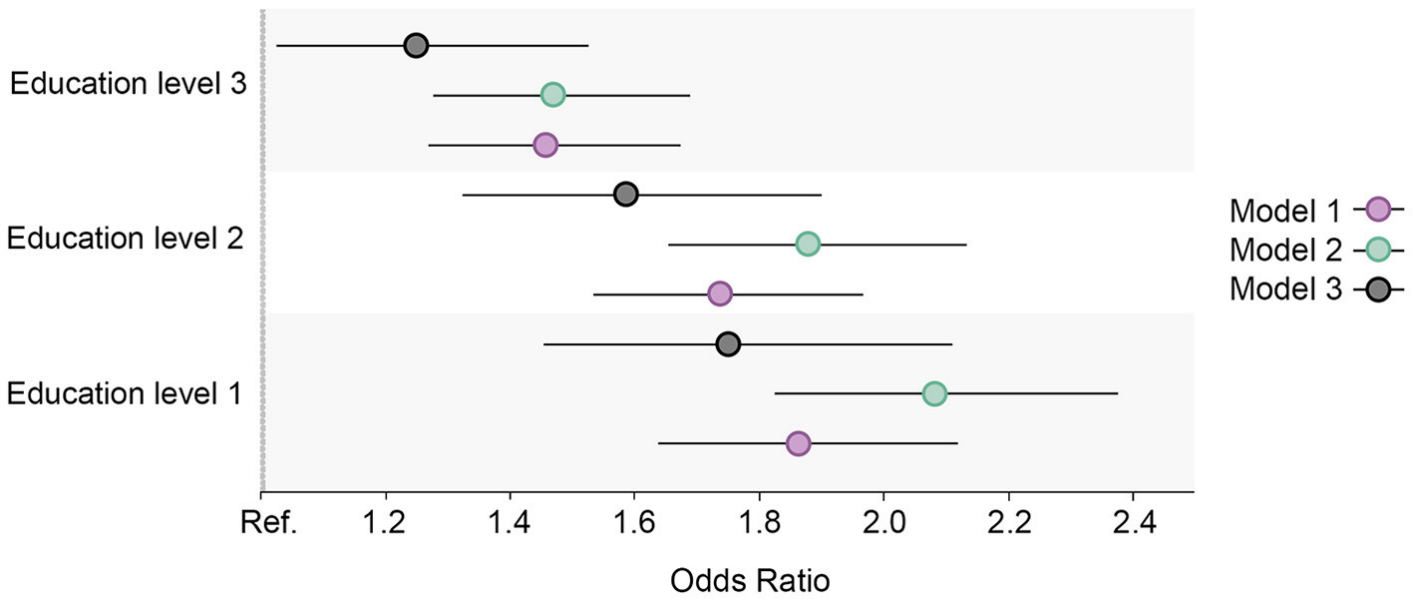
Educational differences in abnormal BMI in the total cohort sample, adjusted for age and sex. Model 1: Effect of education on abnormal BMI at the baseline; Model 2: Effect of education on abnormal BMI at the follow-up; Model 3: Effect of education on abnormal BMI at the follow-up controlled for abnormal BMI at baseline. Education level 1: Primary/partly secondary education; Education level 2: Upper secondary education; Education level 3: University (<4 years); Education level 4: University (more than 4 years). Reference category: Education level 4 [university (4 years or more)].

The prevalence of having a BMI outside of the normal range increased in all education groups and was highest among the two lowest education levels at both the baseline and the follow-up (Figure 1). The prevalence also remained quite high (>65%). The differences followed a clear gradient pattern at both measurement points as shown in the regression output (Figure 5), where the OR increased gradually with a lower education level. The size of the ORs was similar for the third education level at both time points, whereas the estimates increased for the two lowest education levels. When controlling for abnormal BMI at the baseline, a clear gradient was shown, indicating increased educational inequality in abnormal BMI between the baseline and the follow-up.

#### 3.1.5. Healthy lifestyle

**Figure 6:**
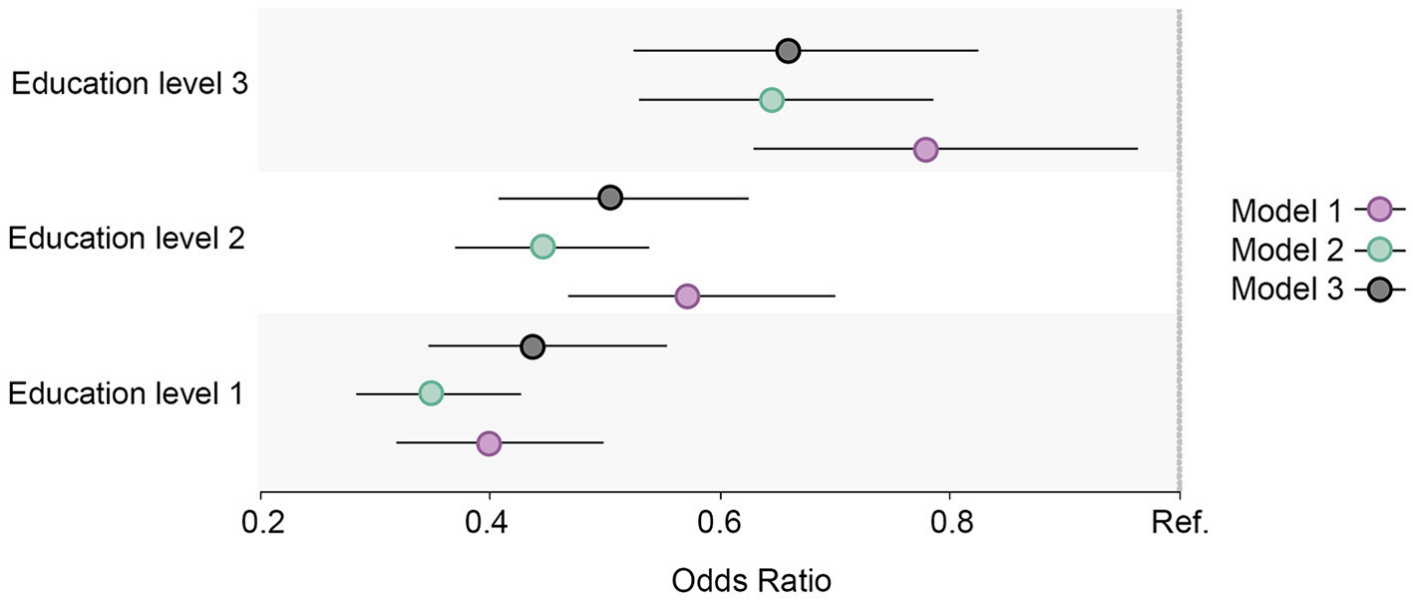
Educational differences in healthy lifestyle in the total cohort sample, adjusted for age and sex. Model 1: Effect of education on healthy lifestyle at the baseline; Model 2: Effect of education on healthy lifestyle at the follow-up; Model 3: Effect of education on healthy lifestyle at the follow-up controlled for healthy lifestyle at baseline. Education level 1: Primary/partly secondary education; Education level 2: Upper secondary education; Education level 3: University (<4 years); Education level 4: University (more than 4 years). Reference category: Education level 4 [university (4 years or more)].

A large proportion of the respondents (43.4%) had only two healthy factors simultaneously (low alcohol intake and non-daily smoking), and this percentage remained stable at the follow-up. The second largest share of respondents were those having three healthy factors (low alcohol intake, non-daily smoking and normal BMI). The proportion with a healthy lifestyle—which is the share of respondents who simultaneously avoided daily smoking and a high alcohol intake, engaged in 150 min of physical activity or more every week, and had a normal BMI—increased over time (8.6–10.2%) and was highest among respondents with the highest education level at both the baseline and at the follow-up (Figure 1). The OR followed a gradient pattern at both time points, where the size decreased gradually as education levels lowered. At the follow-up, the size of the OR was smaller for the first three education groups when compared to the highest education level. This indicates that the highest education level had a greater increase in respondents with a healthy lifestyle (Figure 6). This was confirmed in the analysis of the follow-up that was controlled for a healthy lifestyle behavior at the baseline (Model 3).

### 3.2. Analysis by sex group

**Figure 7:**
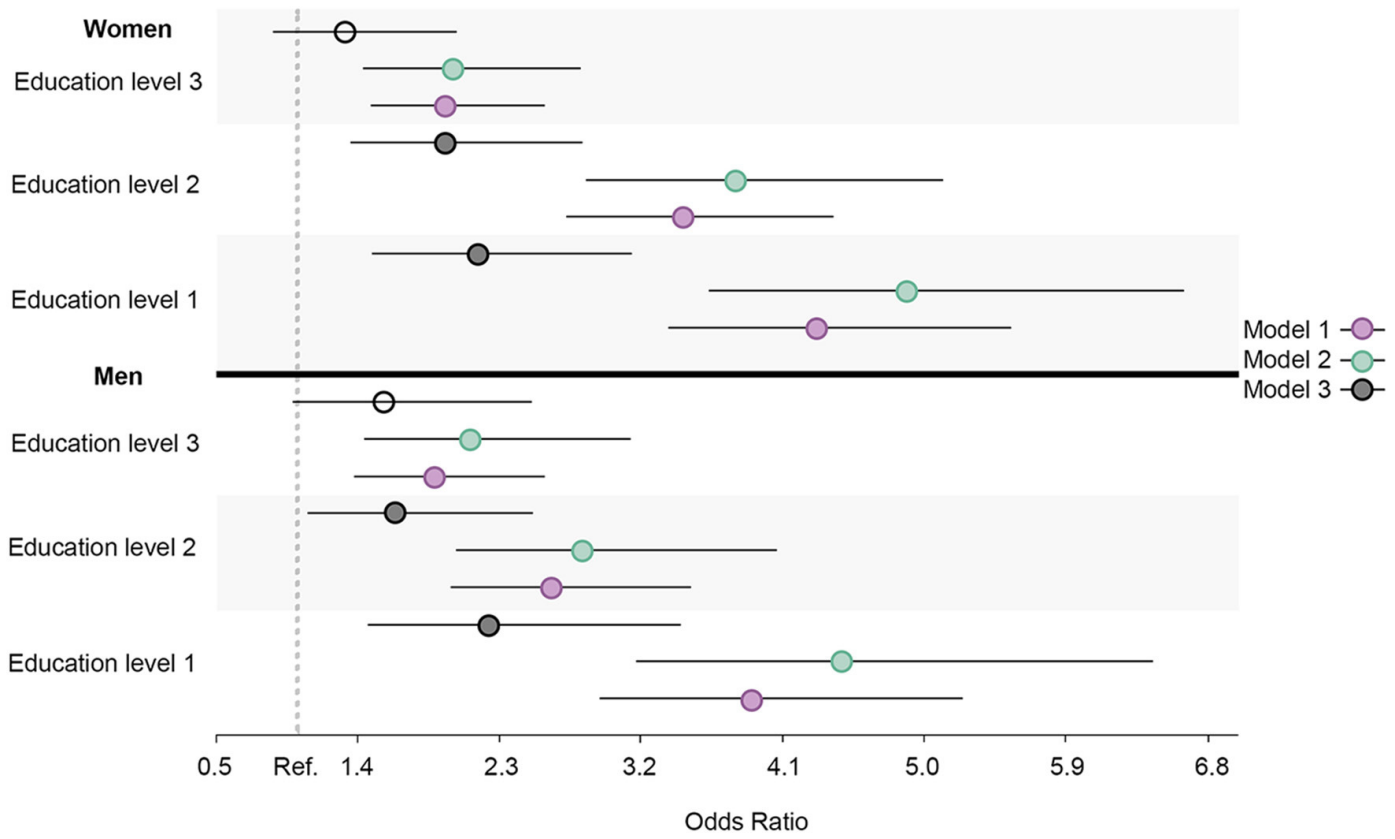
Educational differences in daily smoking among men and women, adjusted for age. Model 1: Effect of education on daily smoking at the baseline; Model 2: Effect of education on daily smoking at the follow-up; Model 3 Effect: of education on daily smoking at the follow-up controlled for daily smoking at baseline. Education level 1: Primary/partly secondary education; Education level 2: Upper secondary education; Education level 3: University (<4 years); Education level 4: University (more than 4 years). Reference category: Education level 4 [university (4 years or more)].

**Figure 8:**
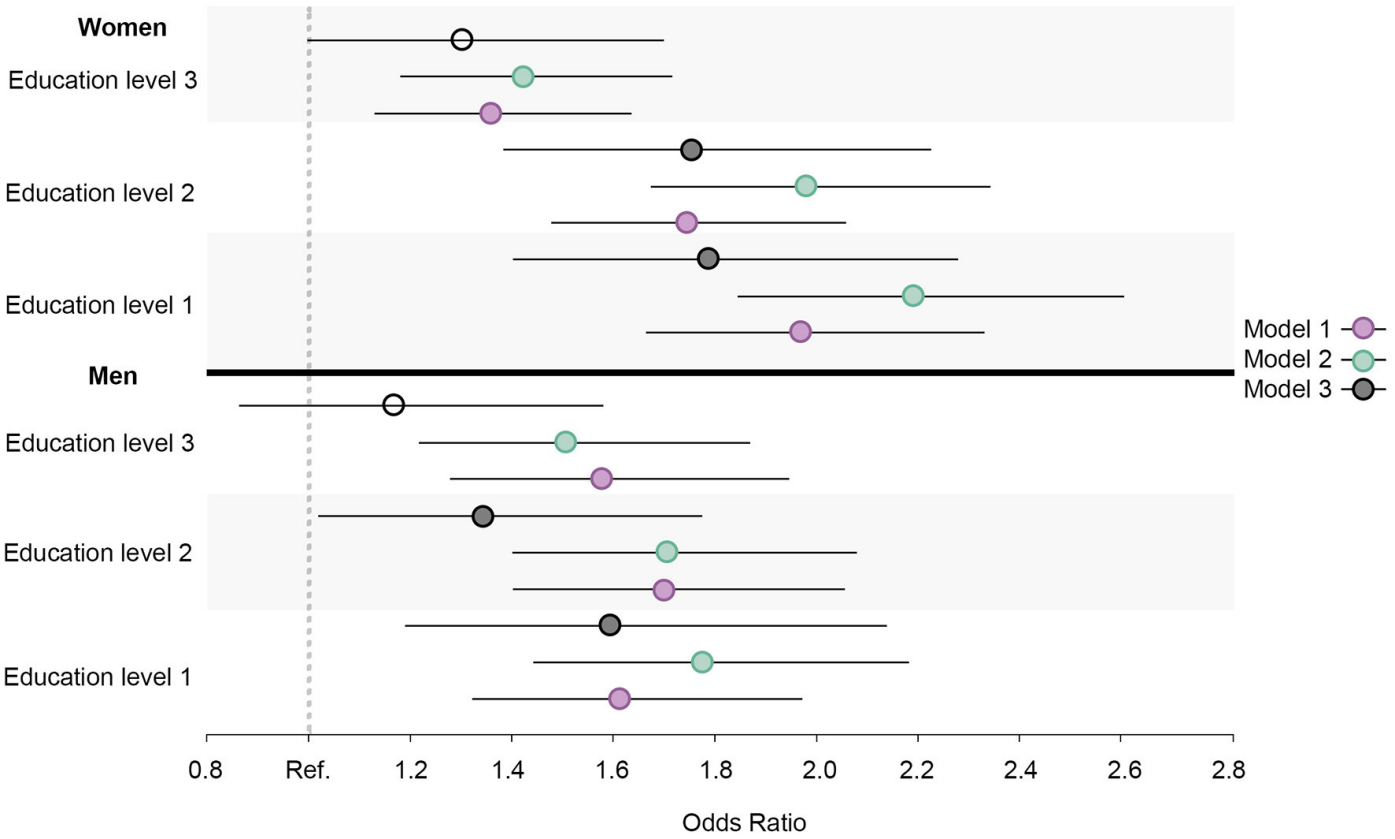
Educational differences in abnormal BMI among men and women, adjusted for age. Model 1: Effect of education on abnormal BMI at the baseline; Model 2: Effect of education on abnormal BMI at the follow-up; Model 3: Effect of education on abnormal BMI at the follow-up controlled for abnormal BMI at baseline. Education level 1: Primary/partly secondary education; Education level 2: Upper secondary education; Education level 3: University (<4 years); Education level 4: University (more than 4 years). Reference category: Education level 4 [university (4 years or more)].

**Figure 9:**
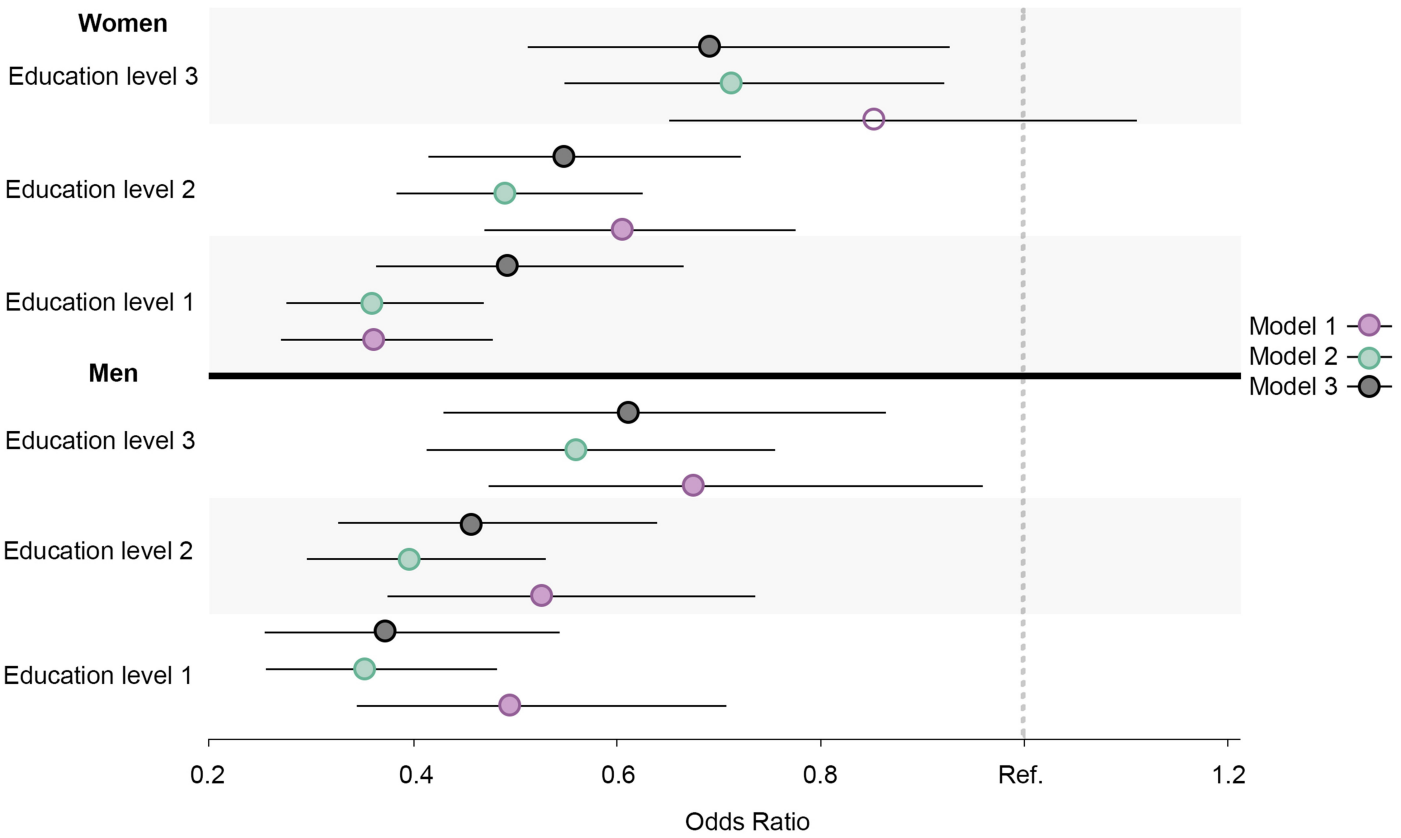
Educational differences in healthy lifestyle among men and women, adjusted for age. Model 1: Effect of education on healthy lifestyle at the baseline; Model 2: Effect of education on healthy lifestyle at the follow-up; Model 3: Effect of education on healthy lifestyle at the follow-up controlled for healthy lifestyle at baseline. Education level 1: Primary/partly secondary education; Education level 2: Upper secondary education; Education level 3: University (<4 years); Education level 4: University (more than 4 years). Reference category: Education level 4 [university (4 years or more)].

**Figure 10:**
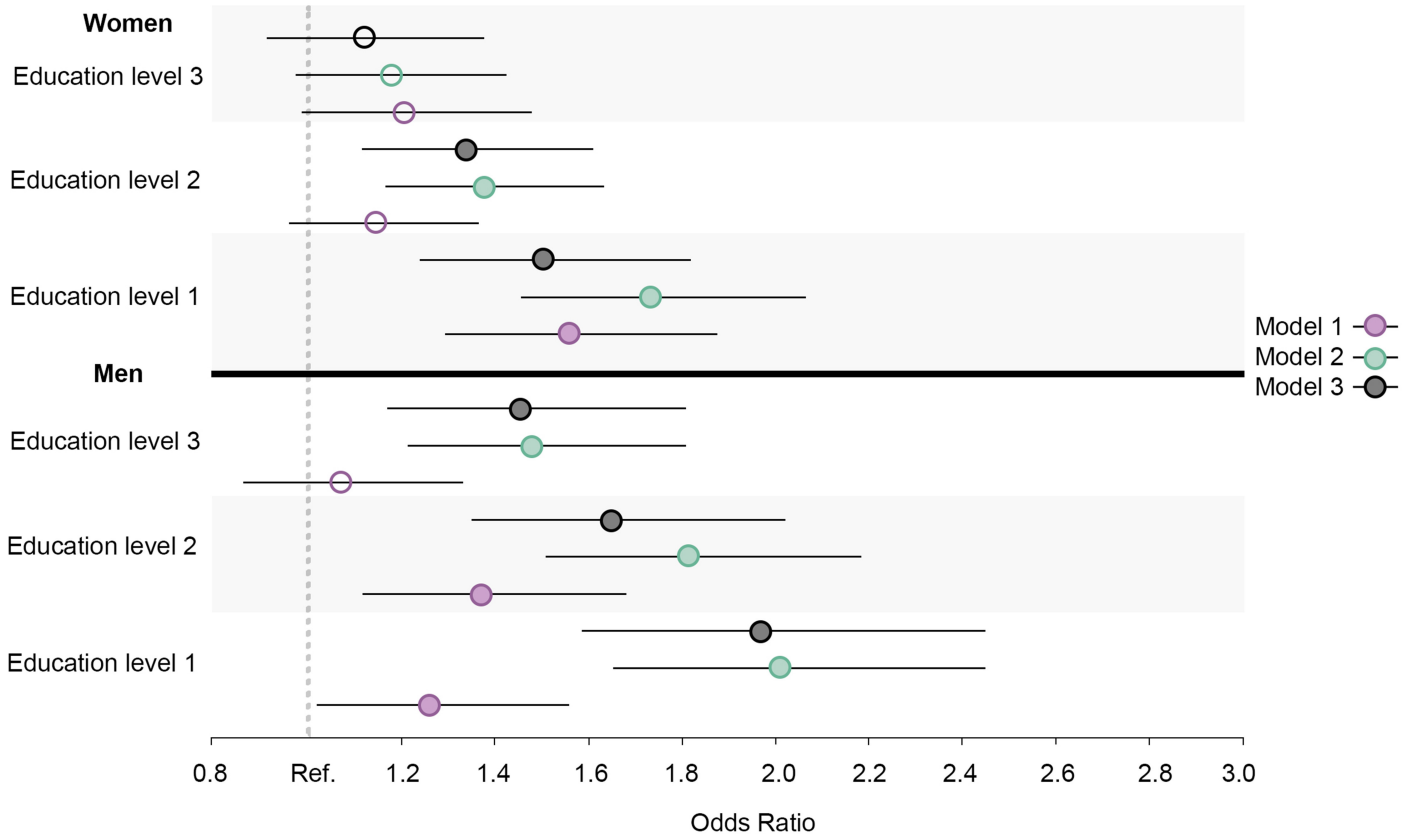
Educational differences in physical inactivity among men and women, adjusted for age. Model 1: Effect of education on physical inactivity at the baseline; Model 2: Effect of education on physical inactivity at the follow-up; Model 3: Effect of education on physical inactivity at the follow-up controlled for physical inactivity at baseline. Education level 1: Primary/partly secondary education; Education level 2: Upper secondary education; Education level 3: University (<4 years); Education level 4: University (more than 4 years). Reference category: Education level 4 [university (4 years or more)].

**Figure 11:**
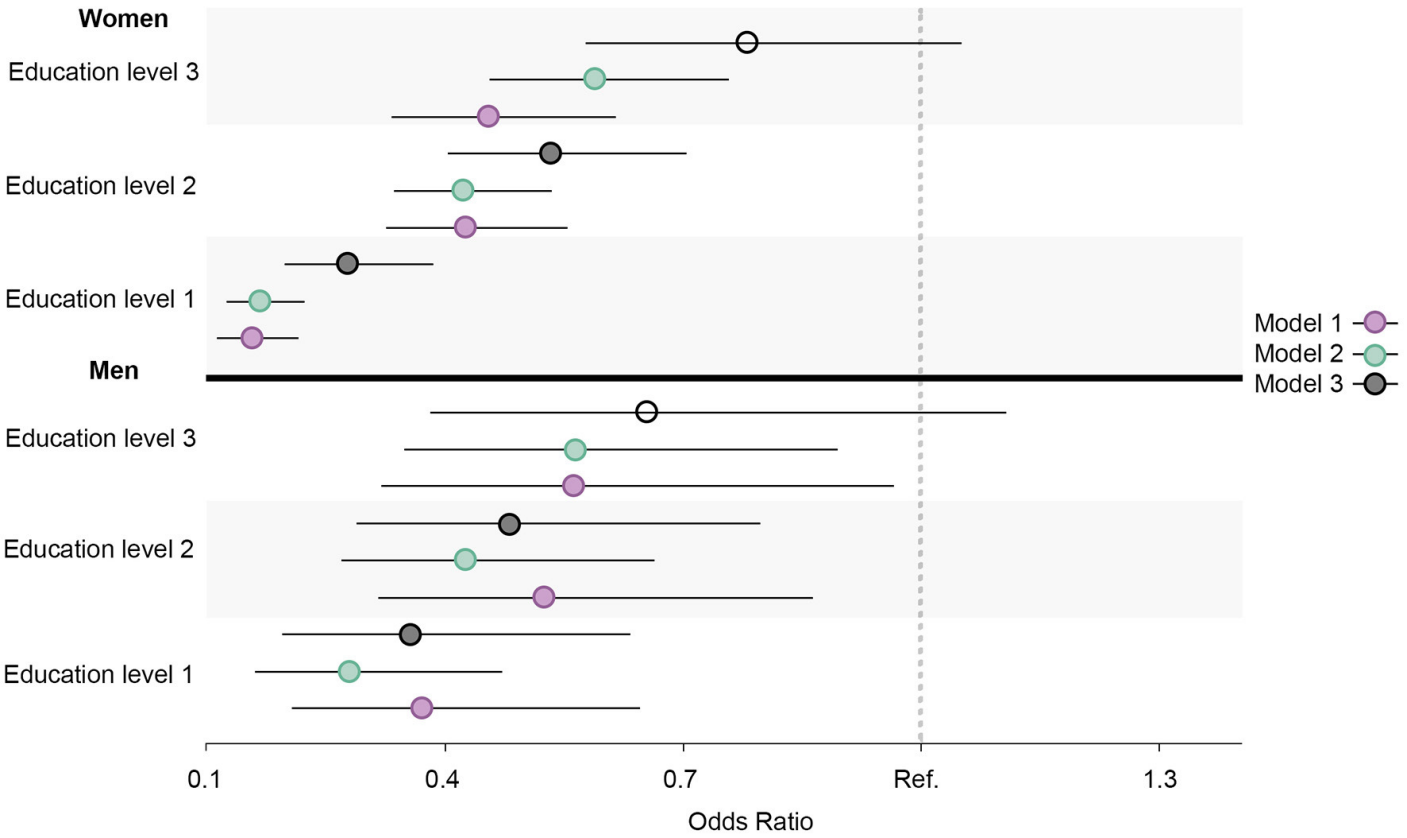
Educational differences in high alcohol intake among men and women, adjusted for age. Model 1: Effect of education on high alcohol intake at the baseline; Model 2: Effect of education on high alcohol intake at the follow-up; Model 3: Effect of education on high alcohol intake at the follow-up controlled for high alcohol intake at baseline. Education level 1: Primary/partly secondary education; Education level 2: Upper secondary education; Education level 3: University (<4 years); Education level 4: University (more than 4 years). Reference category: Education level 4 [university (4 years or more)].

Similar prevalence and OR trends in daily smoking, high alcohol intake, physical inactivity, abnormal BMI, and the healthy lifestyle variable were observed for all education groups in both men and women. When compared to men, the ORs were larger for women in daily smoking (Figure 7), abnormal BMI (Figure 8), and the healthy lifestyle variable (Figure 9), indicating greater relative differences among women than among men in these measurements. In contrast, the physical inactivity ORs were smaller among women when compared to men (Figure 10), indicating larger differences among men in physical inactivity. Finally, high alcohol intake ORs (Figure 11) were also smaller among women when compared to men; however, in this case, the ORs were <1, conversely indicating that differences in high alcohol intake were larger among women. Men and women have different cut-off points for each to fall into the category of high alcohol intake, which partially explains the differences in high alcohol intake trends between sex groups. Additionally, the differences in high alcohol intake between the two highest education levels among women seemed to decrease, whereas the ORs for men followed a reverse gradient pattern at both time points.

**Figure 12:**
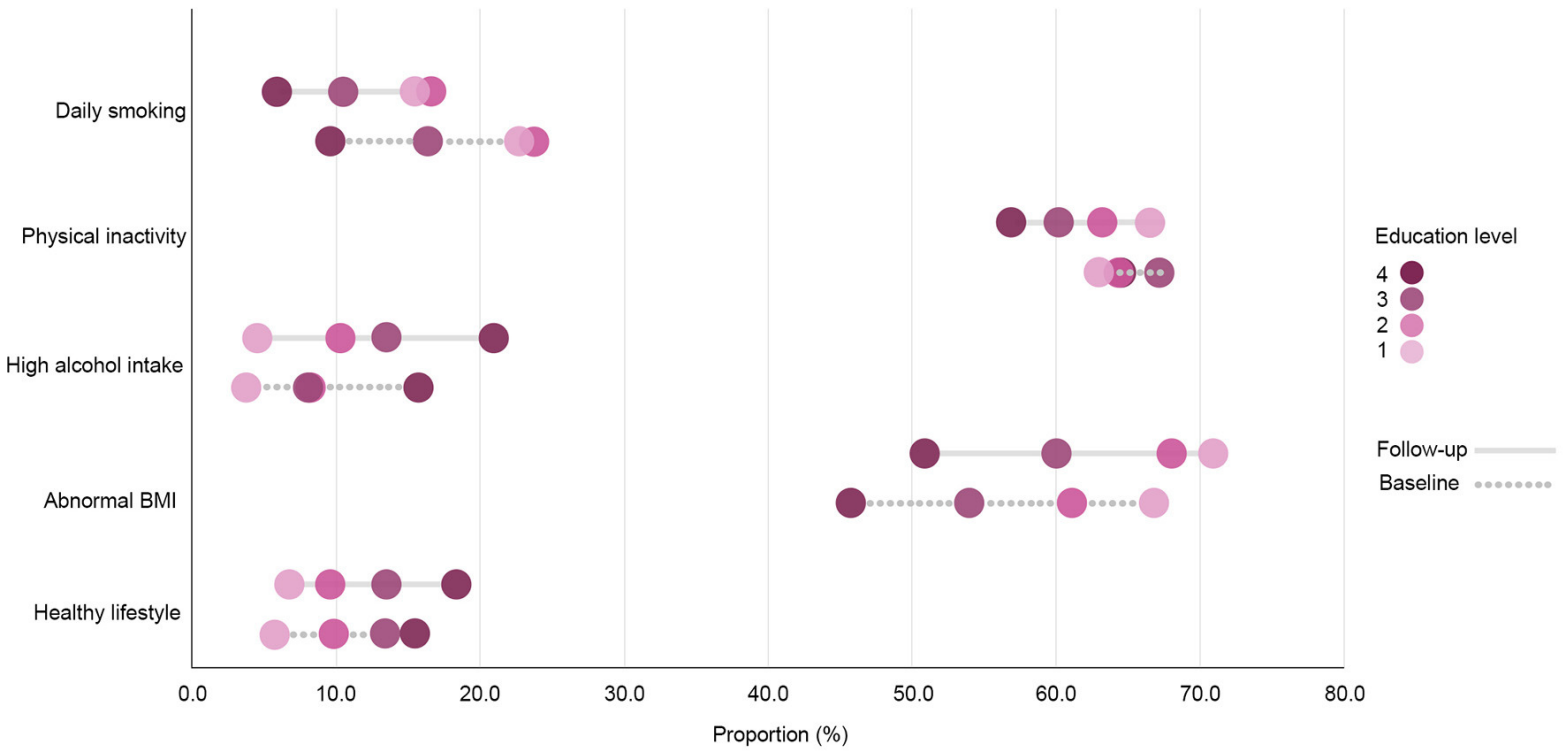
Prevalence rates of health behavior variables among women (*n* = 4,776) by education level at the baseline and the follow-up. Education level 1: Primary/partly secondary education; Education level 2: Upper secondary education; Education level 3: University (<4 years); Education level 4: University (more than 4 years).

**Figure 13:**
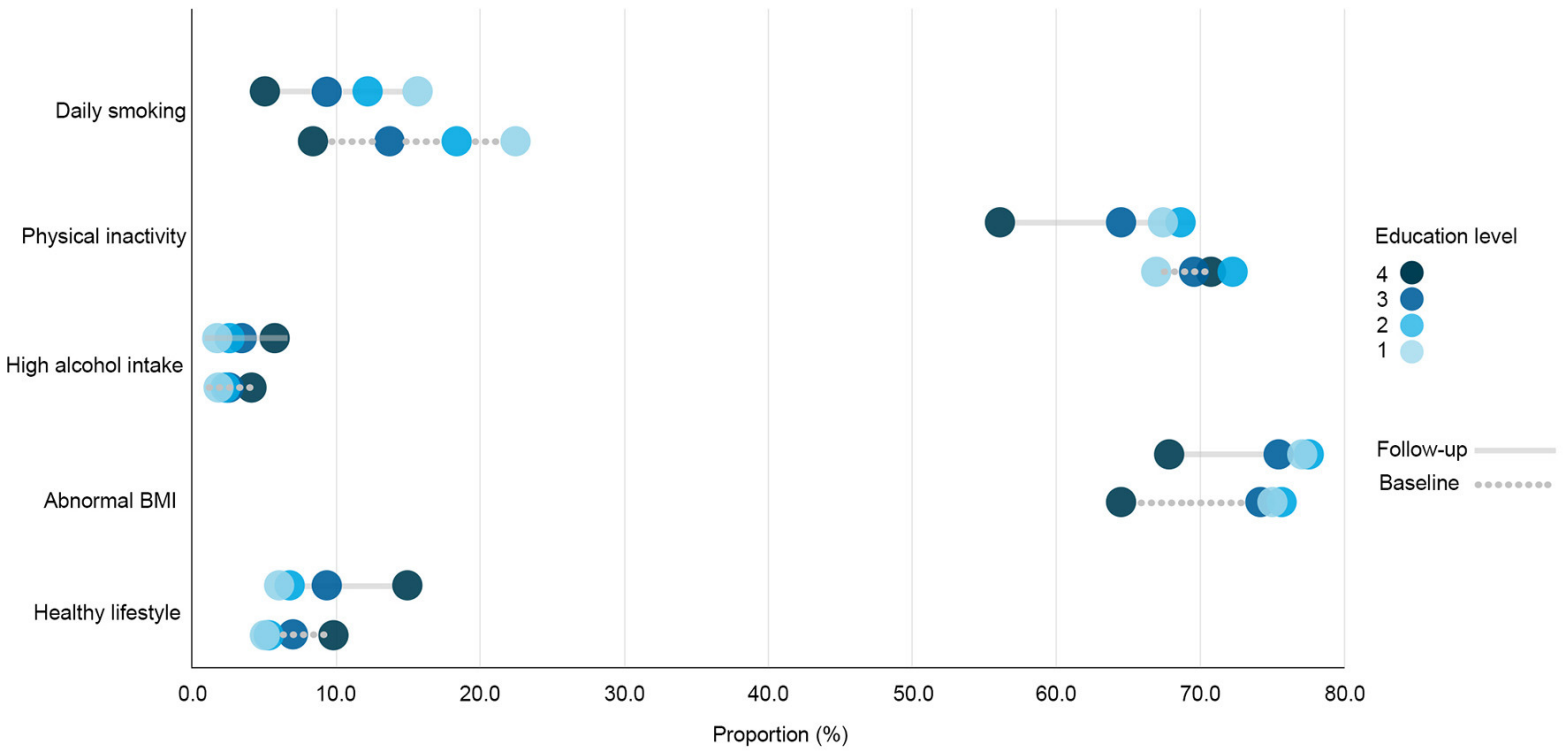
Prevalence rates of health behavior variables among men (*n* = 4,130) by education level at baseline and follow-up. Education level 1: Primary/partly secondary education; Education level 2: Upper secondary education; Education level 3: University (<4 years); Education level 4: University (more than 4 years).

Regarding physical inactivity, there was a clear gender gap in the prevalence of physical inactivity at the baseline, where the proportion among men exceeded 70% (Figures 12, 13). At the follow-up, the highest education level among men and the two highest education levels among women were the groups that increased their physical activity at the follow-up. Over time, there was a considerable increase in inequality in physical inactivity between education levels among men, which was more pronounced than it was for women. Regarding BMI, education's effect on abnormal BMI increased more for women than for men, contrary to physical activity. For men, the change in smoking behavior is similar in the second and third education levels, and the first level of education seemed to engage less in daily smoking than the second and third education levels, whereas the fourth level of education engaged in daily smoking more than the other three education levels. For women, however, changes in daily smoking behavior seemed relatively similar in the two lowest education groups. Regarding the healthy lifestyle variable, the clearest trend observed was that the highest education level is distancing itself further from the other three groups, particularly among women.

## 4. Discussion

Various studies have monitored socioeconomic inequalities in multiple health behaviors using repeated cross-sectional data (35–38). Nonetheless, fewer longitudinal studies have researched changes in socioeconomic inequality in multiple health behavior factors by following the same individuals over time (39, 40). The aim of this study has been to research the changes in relative educational inequality in multiple factors related to health behavior by analyzing the same sample of individuals 8 years apart. Our findings suggest that relative educational differences between the highest education level and lower education levels increased in all measurements related to health behavior. The larger size of the ORs in daily smoking, physical inactivity, and abnormal BMI; and the smaller size of the ORs in high alcohol intake and the healthy lifestyle variable estimated at the follow-up, indicated that the differences between the highest level of education and the other three lower education levels widened from baseline to the follow-up. Similar observations appeared in the analyses by sex group, although the size of the ORs was larger for women than for men in all the health behavior related variables except for physical inactivity. This indicates that, except for physical inactivity, the relative educational differences were larger among women than men.

A similar widening of relative socioeconomic inequalities in multiple health behavior factors has also been reported in previous longitudinal follow-up studies, with larger differences among female adolescents when compared to male adolescents (39). However, another longitudinal follow-up study has shown distinct trends in relative socioeconomic differences between sex groups for multiple health behavior factors (40). For instance, in the latter study, while there was a decrease in socioeconomic disparities in smoking among boys, the opposite was observed among girls. In that same study, socioeconomic differences in alcohol intake were not found among boys, yet, among girls, the reverse socioeconomic gradient found at the baseline seemed to disappear at the follow-up. In terms of physical inactivity, there was only an increase in socioeconomic disparities among boys and not among girls. Our findings suggest that there was an increase in relative educational inequality in the measure of physical inactivity in both men and women, and this increase was more pronounced for men. First, the inferences about gender heterogeneity in health inequalities are sensitive to the health outcome studied and the choice of inequality measure (41). Second, the explanations for gender differences in socioeconomic health inequalities are likely to vary according to life-stage health measures (42). Since the highest level of education was used as the reference group category in our study, the stronger gradient found among women (except for physical inactivity), indicates a larger variation in the prevalence of the health behavior measures between the highest educated group and the three other lower education groups when compared to men. This suggests that attainment of the highest education level may have a greater impact on women's exposure to both unhealthy behaviors and multiple health recommendations simultaneously than it does for men. The exception was physical inactivity, in which the differences between the highest education level and the other three lower education levels were larger among men than women at both baseline and follow-up. In Sweden, a larger relative socioeconomic inequality in physical activity among adult women when compared to men has been observed (43). However, the study did not include potential explanations for the gender discrepancy found in physical inactivity inequality. Other studies on socioeconomic inequality in diverse health mediators that included health behaviors among Scandinavian populations have suggested possible explanations for the sex heterogeneity in socioeconomic health inequalities. For example, a study using Norwegian data suggested that the socioeconomic position that results from establishing household partnerships or couples is a potential explanation for the sex differences in socioeconomic inequalities in diverse health mediators (44).

Regarding prevalence, our findings showed a downward trend in daily smoking and physical inactivity, and an upward trend regarding high alcohol intake, having a BMI outside of the normal range and the healthy lifestyle variable. Despite these changes, the prevalence of physical inactivity and abnormal BMI remained quite high in the total study sample. In terms of the healthy lifestyle variable, while the prevalence increased, it remained low, only slightly above 10% in the total study sample at the follow-up. The rates of practicing multiple healthy behaviors reported in numerous European countries are quite low: few Europeans practice four or more healthy behaviors, with the highest rates reported in the United Kingdom (8.6%) and Finland (9.2%) (45). Moreover, our results showed that not only are there widening relative educational differences in multiple factors related to health behavior separately and collectively, but these differences seem to follow a gradient pattern at both the baseline and the follow-up. The social gradient in health is a well-known phenomenon, which has been constantly reported for a wide range of health mediators and outcomes (46–48). It refers not only to the unequal health distribution between the most and least advantaged population groups but also to how health inequalities tend to form a gradient pattern: a gradual improvement in health with increasing socioeconomic position. While the association between education and health has been extensively explored in the existing research literature, Norway presents a compelling context for further investigating the social gradient in health. The availability of free higher education and the comparatively smaller salary differences (49) suggests that educational disparities in health may be influenced by other factors beyond socioeconomic inequality.

Our study's findings show that the magnitude of both the prevalence rates and the ORs changed gradually with education level. This observation provides valuable insight into the risk that increases with exposure to increasing attainment of education, including its lasting effect over time, which can improve the understanding of the well-known relationship between education and health. The gradient patterning of health mediators and outcomes is key to understanding inequalities in health and assessing the overall returns to education (50, 51).

Our study's findings must be considered in light of its limitations. For example, in terms of selection bias, our study sample was comprised of the individuals who participated in both the sixth and seventh waves of the Tromsø Study, which provided their health behavior measurements for both 2008 and 2016. While this might give a hint of attrition bias, research also suggests that there is little evidence of older respondents introducing additional bias at the follow-up compared to that present at baseline (52). In addition, each analysis excluded respondents with missing data in any of the studied variables, which might suggest selection bias in terms of health behavior and education, given the relationship between lower socioeconomic position and underreporting in health surveys (53). An additional limitation is that the health behavior and education variables were based on self-reported data, apart from BMI, which was calculated from the respondents' height and weight measured objectively at the time of each survey. Nonetheless, information on the education variable in the latest waves of the Tromsø Study was recently validated by Vo et al. (54). In terms of measurements related to health behavior, while current health guidelines provide specifics on a healthy diet, diet was not included in our study due to the major limitations of assessing dietary intake through health surveys (55). Similarly, the variable for physical inactivity was based only on information about frequency and duration, although specific intensities of physical activity are also recommended in current health guidelines (56). Regarding smoking, the respondents who previously had a history of daily smoking patterns were categorized as non-daily smokers. This may potentially introduce bias due to the association between smoking cessation and a higher risk of chronic diseases reported in previous studies (57). Furthermore, all the variables related to health behavior were dichotomized to assess whether the respondents adhere to the specific cut-off points set out by health recommendations. The dichotomization of data has several disadvantages, including loss of information and reduced statistical power (58).

Another possible limitation of the study is that it included all respondents regardless of whether they already had a chronic disease. This could have influenced the results because the presence of chronic diseases may impact individuals' health behavior or perceptions of it. For instance, recent research has shown that participants with lower socioeconomic conditions are more likely to initiate physical inactivity after chronic disease diagnosis when compared to participants with higher socioeconomic position (59). However, that same study also revealed that smoking and physical inactivity were not significantly affected by the onset of chronic disease among individuals with low socioeconomic positions. The findings in our study showed that the group with the lowest education level was the only group in which the prevalence of physical inactivity increased at the follow-up. This trend may be attributed to the onset of chronic disease that may have impaired their ability to engage in physical activity.

Furthermore, regarding the measure of inequality, while OR is a good measure of association and an appropriate relative measure of health inequality (60, 61), problems may arise when OR trends are used in data in which the outcome variable is of relatively high prevalence (i.e., >10%) and varies significantly over time, due to the exponential nature of odds against prevalence (62–64). In addition, different conclusions can be drawn about trends in socioeconomic inequalities in health depending on the choice of relative and absolute measurements (64, 65).

Notwithstanding, the strengths of our study include the size of our study sample, the stratification of analyses by sex group, and the longitudinal design. By analyzing the sample of respondents with repeated measurements, inequality was measured without having to account for population shifts over time (i.e., changes in the distribution of education groups), and thus simple measurements of inequality such as ORs by logistic regression could be used as an efficient measurement of inequality (60). Inequality trends assessed through longitudinal follow-up studies provide information about the timing, duration, and trajectory of changes, which can inform interventions and policies aimed at reducing inequality (66).

As with all epidemiological research, the findings are primarily generalizable to the population from which the data are gathered (67, 68). Tromsø is a municipality and also the largest town in the North of Norway, with a diverse and increasing population (69). It is a substantial rural area with both fisheries and land-based farms, and is among the 10 largest cities in the country. Hence, Tromsø may be considered relatively representative of Norway in general and, to some extent, other countries in Scandinavia. Nonetheless, further studies are required to determine the degree of generalizability of our findings to other countries.

## 5. Conclusion

In conclusion, these findings reveal the extent, persistence, and widening of educational disparities in various factors related to health behavior, and thus associated with chronic diseases. Moreover, explanations for the gender variation in relative educational inequality in multiple health behaviors requires further attention. The unequal distribution of unhealthy behavior factors and inequalities that widen by education level may have implications for the incidence and prevalence of chronic diseases, particularly among population groups with lower education. Understanding and addressing social inequalities in health behavior is crucial to reducing the burden of chronic diseases and promoting health equity.

## Data availability statement

The datasets presented in this article are not readily available due to the presence of potentially sensitive or identifying information regarding the participants of the Tromsø Study. However, information regarding how to contact the Tromsø Study can be found via the following link: https://uit.no/research/tromsostudy. Requests to access the datasets should be directed to Sameline Grimsgaard, sameline.grimsgaard@uit.no; Ola Løvsletten, ola.lovsletten@uit.no.

## Ethics statement

The studies involving human participants were reviewed and approved by Regional Committees for Medical and Health Research Ethics (REK; ID: REK 2019/607). The patients/participants provided their written informed consent to participate in this study.

## Author contributions

AI-S contributed to the conception and design of the study, analyzed the data, interpreted the results, and drafted the manuscript. GC provided guidance on the conception of the study, methodology, interpretation of results, and contributed to editing and reviewing the manuscript. TW provided the overall leadership and guidance to the article, including the conception and design of the study, methodology, data interpretation, and writing and editing of the manuscript. All authors contributed to the article and approved the submitted version.
