# Supplementary material for: Are relative educational inequalities in multiple health behaviors widening? A longitudinal study of middle-aged adults in Northern Norway
Source: Front Public Health. 2023 Aug 22;11:1190087. doi: 10.3389/fpubh.2023.1190087 (PMC10477439; doi:10.3389/fpubh.2023.1190087)
Supplement: Supplementary file 1 [file Table_1.DOCX]

Supplementary Material

Are Relative Educational Inequalities in Multiple Health Behaviors Widening? A Longitudinal Study of Middle-Aged Adults in Northern Norway

**Ana Silvia Ibarra-Sanchez ^1^*, Gang Chen ^2^, Torbjørn Wisløff ^3^**

*** Correspondence:** Ana Silvia Ibarra-Sanchez: ana.s.sanchez@uit.no

# Supplementary Figures and Tables

## Supplementary Tables

|  |  | Total N (%) | | | |  | Men n (%) | | | |  | Women n (%) | | | |
| --- | --- | --- | --- | --- | --- | --- | --- | --- | --- | --- | --- | --- | --- | --- | --- |
|  |  | Baseline | | Follow-up | |  | Baseline | | Follow-up | |  | Baseline | | Follow-up | |
| Total study sample |  | 8906 (100.0) | | | |  | 4130 (46.4) | | | |  | 4776 (53.6) | | | |
| Age mean (range) |  | 55.8 (32-87) | | 63.8 (40-95) | |  | 56.1 (32-87) | | 64.1 (40-95) | |  | 55.5 (32-87) | | 63.5 (40-95) | |
|  |  |  | | | |  |  | | | |  |  | | | |
| Education level | Primary/partly secondary | 2662 | | (29.9) | |  | 1101 | | (26.7) | |  | 1561 | | (32.7) | |
|  | Upper secondary | 2443 | | (27.4) | |  | 1242 | | (30.1) | |  | 1201 | | (25.1) | |
|  | University (less than 4 yrs.) | 1564 | | (17.6) | |  | 834 | | (20.2) | |  | 730 | | (15.3) | |
|  | University (more than 4 yrs.) | 1992 | | (22.4) | |  | 839 | | (20.3) | |  | 1153 | | (24.1) | |
|  | Missing | 245 | | (2.8) | |  | 114 | | (2.8) | |  | 131 | | (2.7) | |
|  | | | | | | | | | | | | | | | |
| Smoking | Daily smoking | 1583 | (17.8) | 1063 | (11.9) |  | 686 | (16.6) | 463 | (11.2) |  | 897 | (18.8) | 600 | (12.6) |
|  | Non-daily smoking | 7231 | (81.2) | 7748 | (87.0) |  | 3413 | (82.6) | 3625 | (87.8) |  | 3818 | (79.9) | 4123 | (86.3) |
|  | Missing | 92 | (1.0) | 95 | (1.1) |  | 31 | (0.8) | 42 | (1.0) |  | 61 | (1.3) | 53 | (1.1) |
|  | | | | | | | | | | | | | | | |
| Alcohol intake^a^ | High | 512 | (5.7) | 675 | (7.6) |  | 106 | (2.6) | 134 | (3.2) |  | 406 | (8.5) | 541 | (11.3) |
|  | Low | 8222 | (92.3) | 8022 | (90.1) |  | 3969 | (96.1) | 3915 | (94.8) |  | 4253 | (89.0) | 4107 | (86.0) |
|  | Missing | 172 | (1.9) | 209 | (2.3) |  | 55 | (1.3) | 81 | (2.0) |  | 117 | (2.4) | 128 | (2.7) |
|  | | | | | | | | | | | | | | | |
| Physical activity | < 150 min/week | 5931 | (66.6) | 5648 | (63.4) |  | 2876 | (69.6) | 2677 | (64.8) |  | 3055 | (64.0) | 2971 | (62.2) |
|  | >=150 min/week | 2347 | (26.4) | 2976 | (33.4) |  | 993 | (24.0) | 1333 | (32.3) |  | 1354 | (28.4) | 1643 | (34.4) |
|  | Missing | 628 | (7.1) | 282 | (3.2) |  | 261 | (6.3) | 120 | (2.9) |  | 367 | (7.7) | 162 | (3.4) |
|  | | | | | | | | | | | | | | | |
| BMI (kg/m^2^)^b^ | Abnormal^c^ | 5795 | (65.1) | 6128 | (68.8) |  | 3008 | (72.8) | 3091 | (74.8) |  | 2787 | (58.4) | 3037 | (63.6) |
|  | Normal^d^ | 3105 | (34.9) | 2747 | (30.8) |  | 1122 | (27.2) | 1029 | (24.9) |  | 1983 | (41.5) | 1718 | (36.0) |
|  | Missing | 6 | (0.1) | 31 | (0.3) |  | 0 | 0.0 | 10 | (0.2) |  | 6 | (0.1) | 21 | (0.4) |
|  | | | | | | | | | | | | | | | |
| No. of healthy behavior factors^e^ | 4 | 767 | (8.6) | 904 | (10.2) |  | 273 | (6.6) | 365 | (8.8) |  | 494 | (10.3) | 539 | (11.3) |
|  | 3 | 2630 | (29.5) | 2827 | (31.7) |  | 1167 | (28.3) | 1315 | (31.8) |  | 1463 | (30.6) | 1512 | (31.7) |
|  | 2 | 3866 | (43.4) | 3864 | (43.4) |  | 1988 | (48.1) | 1901 | (46.0) |  | 1878 | (39.3) | 1963 | (41.1) |
|  | 1 | 796 | (8.9) | 718 | (8.1) |  | 378 | (9.2) | 313 | (7.6) |  | 418 | (8.8) | 405 | (8.5) |
|  | 0 | 38 | (0.4) | 45 | (0.5) |  | 13 | (0.3) | 10 | (0.2) |  | 25 | (0.5) | 35 | (0.7) |
|  | Missing | 809 | (9.1) | 548 | (6.2) |  | 311 | (7.5) | 226 | (5.5) |  | 498 | (10.4) | 322 | (6.7) |

**Supplementary Table 1.** **Characteristics of participants in the total cohort study sample and characteristics stratified by sex group.**
(A) High alcohol intake: more than fourteen units per week for men and seven units per week for women.
(B) BMI: body mass index.
(C) Abnormal: BMI outside of the normal range (under 18.5 kg/m^2^ or more than 24.9 kg/m^2^).
(D) Normal: BMI between 18.5 to 24.9 kg/m^2^.
(E) Number of healthy behaviors: count of healthy behavior factors in every respondent (non-daily smoking, low alcohol intake, >=150 min. of physical activity every week or having a normal BMI).
